# Facts and Sensibilities: What Is a Psychoanalytic Innovation?

**DOI:** 10.3389/fpsyg.2019.01781

**Published:** 2019-08-21

**Authors:** Aner Govrin

**Affiliations:** The Program for Hermenutics and Cultural Studies, Bar-Ilan University, Ramat Gan, Israel

**Keywords:** history of psychoanalysis, philosophy of science, psychoanalytic theories, neuropsychoanalysis, infant research, relational psychoanalytic theory, psychoanalytic developmental theory

## Abstract

Psychoanalytic innovation is easy to recognize but difficult to define. There is a dearth of literature exploring the nature of innovation in our field. My main thesis is that psychoanalytic innovation can be of two types. Psychoanalytic innovation of the first order is about new discoveries concerning facts related to the psyche, development, transference relations, or psychopathology. It usually emerges as a development of insights from canonical psychoanalytic theory; offers an original explanation for a choice of empirical psychic phenomena hitherto unexamined; is perceived as creative and useful when it succeeds to reconceptualize the relations between the patient’s past, unconscious dynamics, and the transference relations; often resembles poetic expression; and registers a truth we knew but did not yet put into words. When it is of the second order, psychoanalytic innovation challenges either methodological or philosophical assumptions held by psychoanalysis, without pretending to replace existing theories. It constitutes a “sensibility” that its adherents strive to incorporate into the existing corpus. I distinguish between two types of sensibilities: cultural -philosophical sensibility represented by the relational approach; and methodological sensibility represented by infant research, and neuropsychoanalysis. In the last part of the paper I analyze psychoanalytic progress pointing to its merits and shortcomings.

## The Problem

Does psychoanalysis progress? And, if so, what does progress in our field mean? Can we find regularities in the last decades of this progress?

Understanding innovation in our field is challenging. On the one hand, psychoanalysis is in constant flux: Those who currently understand their patients according to Freud’s original topographic and structural model are few and far between; members of the professional communities inspired by Klein or Kohut do not work in the same way as their predecessors. Even our understanding of fundamental psychoanalytic concepts such as the unconscious, interpretation, object relations, transference, and countertransference has grown extremely varied.

On the other hand, the past remains extremely influential: The twentieth century’s grand theories are more dominant than any current development. No new school of thinking including a novel developmental theory, therapy, or psychopathology has emerged since the 1980s (for definition of a psychoanalytic school, see Govrin, 2006). Most current innovations are reinterpretations of old, canonical texts.

The most read and cited papers are by psychoanalysts who are no longer alive. Psychoanalysts representing different approaches continue to work in a way that does not stray far from Freud’s method: together with the patient they create a narrative hinging on the latter’s early development and transference relations. It is therefore practically impossible to distinguish between old and new.

### First-Order and Second-Order Questions

We may subdivide the development of psychoanalysis after Freud into two periods. In the first four decades after Freud’s death, there is a downpour of new and rich theories about the human psyche: Klein, Lacan, Winnicott, Bion, Mahler, Hartman, Kernberg, and others. We can call this the, second, “silver era” of psychoanalysis, coming after Freud’s groundbreaking work. They focus on other psychic phenomena, have a different understanding of the transference relations, focus on other psychopathologies, and redraw the map of child development. They see themselves mainly as continuers, part of a line starting with Freud, rather than as revolutionaries, and they either compete with each other in terms of their adherence to Freud (Klein, Freud, Hartman) or work hard to show that they continue his theory in some way but differ in others (Kohut, Winnicott).

During this period, there is no declared change in philosophical and methodological assumptions. Clinical practice is revolutionary and seething with debate, but the methodological foundation stays as it was: it relies on clinical psychoanalytic observation in the development and construction of psychoanalytic theory. The prevailing truth is correspondence with reality, i.e., realism: The therapist is the authority; the distinction between subject and object remains in place; and developmental theories and psychic structures are considered mental realities rather than useful metaphors.

In this domain, debates are like those between scientists: Are the assumptions underlying the innovation valid or can they be refuted?

The arrival of postmodernism signals a breaking point and ushers in the third era. Self-psychology, in the 1980s, is the last major psychoanalytic school, or Grand theory, a mega narrative that encompass all mental phenomena.

From this point onward, there are two types of questions that analysts deal with: second and first order. The already existing schools of Lacan, Bion, Klein, and Winnicott continue to find explanations to first-order questions. They develop within the existing schools the meaning of psychic occurring such as envy, anxiety, sexuality, the unconscious, psychopathology, and transference phenomena (for a description of innovations of each school, see Govrin and Mills, 2019).

Second order claims are made about knowledge itself. If psychic phenomena involve first-order empirical questions about the substantive entities of the “mental”, second-order questions concern the internal consistency, the methodology, or the epistemology on which first-order questions rely (Laudan, 1977). Why are there slips of the tongue is a first-order empirical question, the “fact” that it reveals an unconscious thought is a first-order explanation. However, what is the best way to validate our explanation about slips of tongue is a second order question. Note that the first-order question such as “why are there slips of the tongue?” is independent of its explanation. It does not need to be phrased in a psychoanalytic jargon. It is inseparable from Freud’s repression theory, and therefore, it is not theory-laden. The importance of the difference between first-order questions and theories (explanations) which attempt to solve them will be elaborated later (see also Laudan, 1977, pp. 139–146).

During the last decade, a few psychoanalytic communities incorporate other fields of knowledge to create new sensibilities that deal with second-order questions. There are two kinds of sensibilities: Methodological sensibilities which are scientifically oriented and present a new source of information and methodology. Currently, there are at least two types of methodological sensibilities: Neuropsychoanalysis derives from brain research and infant research which incorporates finding from infant studies to the psychoanalytic encounter (mentalization-based therapy is also a methodological sensibility). The other type is a cultural-philosophical sensibility. It is influenced by changes in society, culture, and philosophy. It adapts psychoanalysis to the changing world. Since the 1990s, this sensibility can be observed in the relational approach, which was inspired by postmodernism, contemporary feminist thinking, and intersubjectivity. Neither of the three sensibilities are meant to be new psychoanalytic schools or alternative psychoanalytic theories. Nevertheless, they do want analysts to be sensitive to aspects of the psychoanalytic encounter that they argue were overlooked. Of the three sensibilities, the relational approach was the most influential with thousands of practitioners joining and establishing a worldwide organization that basically supported a transformation in how analysts perceive the analytic encounter and the analyst’s subjectivity.

In this paper, I investigate innovations in first- and second-order questions. But let us begin with some preliminary remark.

I discuss psychoanalysis in general, even though it is far from being monolithic (English-speaking psychoanalysis is quite different from French or Latin-American psychoanalysis, not to speak about the variety of schools - Freudians, Lacanians, Millerians, Winnicottians, Kleinians, Jungians, etc.). But discussing the innovative aspects of each of these branches would require another paper. This paper does not present an historical description of innovations in psychoanalysis. Its focus is rather on particular revolutionary developments so as to illuminate what novelty in psychoanalysis involves.

## Theoretical Innovations in Psychoanalysis

The Merriam Webster Dictionary offers two definitions for innovation: (1) the introduction of something new and (2) a new idea, method, or device.

For a theoretical innovation to be valuable, it can be expected to creatively improve something that already exists. I will therefore include this sense of usefulness in my notion of innovation, as well as a certain type of thinking outside the box.

How do psychoanalysts decide that something is innovative? Going by the above mentioned definition, psychoanalysts are not likely to agree what counts as innovative in their profession. When Klein presented her new ideas in the field of infant development at the London Institute of Psychoanalysis in the 1940s, she met with the opposition of Anna Freud and her colleagues who considered them as anything but an innovation: They saw them as a foreign body in psychoanalysis and did all they could to keep them out (King and Steiner, 1991). When Kohut introduced empathy as a central concept at a conference in the USA, some major establishment figures left the auditorium (Strozier, 2001). Both Klein and Kohut however created impressive and complex theories, which eventually changed psychoanalysis, and they have come to be seen as significant innovators.

While certain innovations were embraced by a majority of the psychoanalytic community, others were ignored and forgotten. Here are two examples.

First is Klein’s concept of projective identification, which was developed by for instance Bion (1959) and Casement (1985).

It hits the psychoanalytic world like a meteor, leading to substantial changes in the perception of the psychoanalytic encounter. Projective identification prompted a wealth of publications and conferences elaborating it from various perspectives. Its brilliance lies in the same stroke of genius that produced Freud’s notion of transference: Something hitherto regarded an obstacle to treatment transforms overnight into a resource, diagnostic as well as therapeutic. The notion of projective identification enables a new reading of therapeutic processes.

Second, there is innovative psychoanalytic knowledge that has not been well-received by the community, has been rejected and forgotten. An example is the fascinating experimental work of Silverman (Silverman, 1982, 1985) on subliminal psychodynamic activation. Through this method, Silverman attempted to identify the unconscious conflicts associated with specific symptomatic behaviors. Findings indicate that when participants are shown a subliminal message - “Mommy and I are one” - this leads to improved behavior and reduction of a variety of symptoms, from psychotic ones in schizophrenic patients, through nicotine addiction and academic underachievement all the way to phobias. The results of this research impressed psychoanalysts when they came out.

But in spite of its promising reception, the method was not eventually embraced by psychoanalysts and they did not seek to equip their clinics with a tachistoscope. No matter how innovative, subliminal psychodynamic activation never made it into psychoanalytic practice and fell into oblivion.

We may conclude that for an idea to be regarded as innovative, it takes a community to consider it as such. Truly innovative psychoanalysts never worked in isolation: their innovation was the product of an entire network. Obviously, it took genius and profound theoretical knowledge in order to invent notions like transitional object, schizoid-paranoid position, and reverie. But it was even more fundamentally necessary for there to be a tight network of psychoanalysts who were writing about the new conceptualization, elaborating it, employing it in the clinic, discussing it with their supervisees, and lecturing on it in psychoanalytic meetings.

This is why my preferred definition of psychoanalytic innovation crucially refers to the psychoanalytic community: Psychoanalytic innovation is a conceptualization found effective by the psychoanalytic community in yielding a new insight into psychoanalytic technique, clinical, or mental phenomena. An accepted innovation is where a psychoanalytic community finds something that so far eluded its understanding. This innovation must respect psychoanalytic specificity. It cannot be merely effective. Psychoanalysis, to begin with, is a special type of response to a special type of appeal. It rests on an ethics whose products are the results of the patient’s psychic search into their self with as their main tool the therapeutic relationship and the centrality of the unconscious. Psychoanalysis aims not only to relieve suffering or to get rid of symptoms, it holds the fundamental principle of “Know Thyself” – a knowledge which every psychoanalytic approach conceptualizes in its own way.

Now, we are in a better position to understand why we do not find tachistoscopes in analysts’ clinics, but we do find a preoccupation with projective identification, transitional objects, and self-object needs. Silverman’s subliminal psychodynamic activation was not taken up in psychoanalysis precisely because it is very remote from the specificity of psychoanalysis: which aims to find where transference intersects with the patient’s needs and conflicts. It is much closer to hypnosis and suggestion which impose conscious, explicit, and deliberate suggestion on the analysand. This is absolutely anti-psychoanalytical, “useful” as it may be. It is a short way to alleviate symptoms. It is not the psychoanalytic way.

### Is Psychoanalysis Obsolete?

Critics of psychoanalysis argue that the discipline does not move with the times: psychoanalysts often refer to texts that were originally published a century, 80 or 50 years back. In this, they argue, psychoanalysis resembles religion, which refers to a static canon. This is factually undebatable. The Psychoanalytic Electronic Publishing (PEP) online archive comes up with two statistics: the most cited and most viewed articles. We may assume that most frequently read and cited articles reflect what is most influential among contemporary psychoanalysts, what has left most marks on the community, and the authors on whom psychoanalysts rely when they write their own papers. In PEP statistics for the past 5 years, the most frequently quoted article is by Klein, originally published in 1946: “Notes on Some Schizoid Mechanisms” (Klein, 1946). The list of the most frequently accessed articles is headed by an article by Winnicott from 1953: “Transitional Objects and Transitional Phenomena – A Study of the First Not-me Possession” (Winnicott, 1953).

Most papers on the list of the most popular articles were published between 1935 and 1992, and only two of them are from the early 2000s–Ogden and Benjamin. The majority of the best read psychoanalysts are no longer alive: Klein, Ferenczi, Bion, Rosenfeld, Winnicott, Strachey, and Joseph.

This state of affairs suggests two things: First, postmodernism’s great insight about the nature of truth seems hardly to have penetrated the community. A huge proportion of the 30 most accessed and most cited papers are by authors who wrote before the advent of postmodernism (Winnicott, Klein, Joseph, Bick, Sandler, Kohut, Rosenfeld, and Heiman) and represent what relational psychotherapy has come to call a “one person psychology.” This arguably signals a triumph of the old school over postmodern approaches.

Second, this list of dead authors might tempt one to draw the sad conclusion that psychoanalysis is grinding to a standstill; that the community goes on celebrating the achievements of the past; and that none of the articles written since have had a comparable impact to the cherished canonical texts written up to roughly the mid-1980s^1^.

As mentioned, critics of psychoanalysis comment negatively on its tendency to dwell on the past.

As Bornstein and Masling (1998) noted, “A geneticist of 1900 could not sustain a conversation with a contemporary geneticist, but Freud would have no trouble recognizing the psychoanalysis of 1997 or reading a modern psychoanalytic journal” (pp. xviii–xix).

According to Bornstein (2001), psychoanalysis lacks any innovative quality. Uncurious about the extra-analytical scientific world, and communicating only among each other, psychoanalysts do not expose themselves to alternative theories that might enrich their knowledge.

But this criticism mistakes the essence of psychoanalysis when it expects that, like scientific discovery, it will reveal new facts which then replace earlier, superannuated ones. It fails to bring into account that psychoanalysis is a language that deciphers meaning, yields insights about the psyche, insights that can be used again and again.

Psychoanalysis is more like philosophy and literature than like science. While scientific theories in use decades ago will have given way to more novel theories, philosophical theories going back to antiquity, for instance, still continue to inspire, drawing interest among contemporary philosophers. University departments of philosophy around the world teach ancient Greek philosophy all the way to the philosophy of the Modernity – Spinoza, Kant, Mill, Hume, Hegel, Nietzsche to mention but a few. These philosophers continue being read because the depth of their insights transcends historical periods, and their relevance does not diminish. If the type of statistics we found on PEP were to be done on periodicals in philosophy, they would also reveal a high percentage of quotations and viewings of no longer living authors.

The same goes for literature. Netz (2016) provides an illustration from the field of papyrology: In 1896, in Egypt, thousands of decaying papyrus scrolls were discovered. Scholars were excited: new texts and new books in the literature of ancient Greece were about to be discovered, they believed, and the study of Antiquity was about to undergo a great upheaval. Here and there, indeed, an ancient papyrus carrying a hitherto unknown text was found (a poem by Sappho, for instance). But such incidents were rare. By far, the most discovered scrolls were manuscripts of texts we already knew: the same Plato again, and the same Homer. The scholars’ hope that our ancient forefathers knew other writers than the ones who were copied onto parchment in the Middle Ages proved vain. Monks in medieval Constantinople chose to copy the very same texts that had been copied on papyrus, in Egypt, a millennium earlier. It transpired that literary taste does not tend to change and renewal. Sophocles, Aeschylus, and Euripides were Athens’ most popular playwrights even in their own life times. And they remained so for the next 2,500 years.

Conceptualizations concerning the psyche, likewise, do not lose their relevance rapidly. Truths of this type go on being discovered by one generation after another. This type of innovation might be captured by the notion of wisdom, which casts a new light on mental life. And much like in the case of philosophy, these ideas become the source of inspiration for new ideas, which in turn will multiply.

Analysts reread classical references and adapt Freudian or Kleinian or Winnicottian methods to contemporary clinical fields. Innovation comes through interpretive extension.

Three citations from a philosopher of science, a philosopher of aesthetics and a poet illustrate the connection between tradition and innovation in nonpsychoanalytic fields.

Kuhn writes on scientific revolution that it “requires a thoroughgoing commitment to the tradition” with which the fully successful innovator eventually breaks (Kuhn, 1977, p. 235).

Cavell, referring to art, also maintains that radical innovations occur in significant dialogue with the past. Here, he describes radical breaks in the music of twentieth century:

“What looks like ‘breaking with tradition’ in the succession of art is not really that; or is that only after the fact, looking historically or critically; or is that only as a result not as motive: the unheard of appearance of the modern in art is an effort not to break, but to keep faith with tradition” (Cavell, 1976, pp. 206–207).

Eliot wrote: “The poem which is absolutely original is absolutely bad; it is, in the bad sense, ‘subjective’, with no relation to the world to which it appeals” (Pound, 1934, p. x).

## Four Characteristics of Psychoanalytic Innovations of the First Order

Psychoanalytic innovation of the first order (new discoveries concerning facts) has four characteristics.

### First, Most Innovations Concern New Psychic Dynamics Linking Between Transference, Psychopathology, and Infants’ Mental Life

The 30 most cited and viewed papers written by Klein, Winnicott, Bion, and other prominent figures, the psychoanalytic canon, go on inspiring new work, and these prompt us to consider what it is about these inspirational sources that lead to innovative production.

While the innovative production represents different approaches (object relations, self-psychology, and relational psychotherapy), we can observe a common pattern: Almost without exception they tie together the three following domains: development, psychopathology, and therapy. Hardly, any one of them addresses only one of these topics in isolation. Psychoanalysts adhering to the various approaches find such narratives the most useful to their therapeutic work.

The specific psychoanalytic approach of the article in question does not seem to matter: Whether it is self-psychology, Klein, Winnicott, or Ferenczi, the patient’s psychopathology appears in relation to the transference, and the transference is related to early interactions with the caregiver. Canonical articles, it appears, all present the regularities at work between these same components. So tightly interlinked are these three components, that it is enough to ask any psychoanalyst to describe the key stations in an infant’s life to gain a revealing insight into his approach and the way he conceives of transference. And once we know how a psychoanalyst defines transference, we can tell with reasonable accuracy what her ideas about infant development are.

So, if a novel idea in psychoanalysis is to have an impact it should be tied to infantile mental life on the basis of both transference relations and mental distress and to the ability to give new meaning to transference relations on the basis of infantile mental life. Just as a problem in biology is solved in terms of biology not in those of chemistry a psychic fact is understood in this particular psychoanalytic way. This way of thinking has not altered since Freud and is in fact a variation on the transference neurosis, namely, the idea that the patient’s early childhood conflicts resurface in the transference relationship with the psychoanalyst.

That innovations feed off the tradition does by no means reduce their creativity and contribution. Some innovators adhered faithfully to one theory, but were plentifully creative within that context, and their extension of it can be considered significant and very influential. This is the case for instance with Joseph’s work on Klein, Ferro’s on Bion, and Goldberg’s on Kohut, to name just a few. Other authors do not follow one theoretician but refer playfully and creatively to the existing literature, dancing as it were between various theories, and leaving their personal marks in the creative links they forge, together with their own personal additions. Examples of this type of work are for instance by Bollas, Eigen, Phillips, and Milner.

New theoretical development can consist of illustrating an existing psychoanalytic conceptualization (through, for instance, a case study in which the concept is employed for clarification); extensions of a psychoanalytic conceptualization to new populations (e.g., applying self-psychological principles to issues concerning eating disorders); elaborating an existing conceptualization (e.g., manifestations of the female castration complex); discovery of an interesting therapeutic process and illustrating a clinical phenomenon by means of it; and in-depth discussion of the work of an important psychoanalyst, or exposition of a hitherto neglected text by her or him.

### Second, Psychoanalytic Innovation Solves Empirical Problems

Psychoanalysis is a psychology. It addresses clinical phenomena, first-order facts, independent of theory: Patients suffering from hysterical symptoms visited Freud’s clinic and he had no idea what might be wrong with them. Having identified hysteria as a clinical phenomenon, Freud was then able to discover the unconscious, the various other mental structures and eventually to develop his overarching theory.

The philosopher of science Laudan (1977) argues that science fundamentally aims at the solution of problems. He proposes that theories should be evaluated on the basis of how adequately they solve significant problems. It is much less relevant to ask whether they are “true,” “corroborated,” or “well-confirmed.” He claims that one of the main kinds of problems scientific theories want to solve is empirical problem. An empirical problem, he argues, is “anything about the natural world which strikes us as odd or otherwise in need of explanation” (p. 15).

Empirical problems in psychoanalysis may be parapraxes, a 3-year old’s fixation on her doll, intense, destructive jealousy, dreams. All these phenomena exist in the world as they are unrelated to psychoanalytic theory. In fact, human suffering in all its forms and variations constitutes an empirical problem, as well as the ways of treating it.

Some of the most interesting innovations in psychoanalysis have to do not just with solving empirical problems but discovering the problems in the first place. Many psychoanalytic phenomena had first to be recognized as empirical problems or felt to require explanation or clarification prior to these descriptions. Slips of the tongue were well known before Freud but were not considered to be a scientific or empirical problem. Another example is Winnicott’s transitional object. The fact that a toddler clings to a teddy bear was well known before Winnicott gave it an explanation.

Often innovators focus on an existing (nonanalytic) phenomenon and show its relevance to psychoanalytic theory. Hence, I assume that psychoanalytic innovation will, more than anything, occur in the field of empirical problem solving. Regarding first-order questions, innovation means a new explanation (“solution”) to psychic phenomenon (“empirical problem”).

Psychoanalytic journals are simply bursting with such topics. Here are some examples: “The Role of the Nanny in Infant Observation” (Yakeley, 2017); “The Masculine Vaginal: Working With Queer Men’s Embodiment at the Transgender Edge” (Hansbury, 2017); and “The Encounter Between Holocaust Survivors and Perpetrators” (Auerhahn and Laub, 2018).

Another type of problem that theory might address is conceptual in nature. Such problems emerge from the theory itself; they have no existence outside the theoretical field in which they arise. Here are some titles reflecting this:

Projective Identification and Relatedness: A Kleinian Perspective (Roth, 2017); “Comparative Assessment of and Bion and Winnicott’s Clinical Theories” (Aguayo and Lundgren, 2018); Truth Axes and the Transformation of Self (Yadlin-Gadot, 2017).

### Third, Psychoanalytic Innovations Are Formulated Poetically

The new knowledge however is not communicated as if it was, say, medical knowledge. Often texts in this domain seem vague, open to interpretation, hard to understand, and more like poetry in their formulations.

In typical texts by Bion, Ogden, Winnicott, or Eigen, sentences are often ambiguous and labyrinthine, meaning is dense and layered, the opposite of the clear and simple style that scientists admire. Often the reader, in addition to gaining new knowledge or discovering an interesting solution, has an aesthetic experience which involves powerful emotional intensities. They seem to resonate deep layers with which the reader now makes contact for the first time. The echoes of the text, their links, the things they are attentive to, and all the others ways in which texts and objects can be tied, begin to struggle in the reader’s or listener’s mind with the semantic content (Scruton, 2015).

Canonical texts in this sense offer an enigmatic, complex phenomenon demanding explanation in its own right, like poetry. Most of the major psychoanalysts are rather gifted writers, and their texts may resemble literary prose or poetry more than properly scientific texts.

“As regards the beauty of Nature”, writes Freud in On Transience (Freud, 1942/1915) when he was preoccupied with the losses of WWI, “each time it is destroyed by winter it comes again next year, so that in relation to the length of our lives it can in fact be regarded as eternal (Freud, 1942/1915). The beauty of the human form and face vanish forever in the course of our own lives, but their evanescence only lends them a fresh charm. A flower that blossoms only for a single night does not seem to us on that account less lovely” (pp. 304–305).

But psychoanalytic thinking is not poetry and the poet has somewhat different motivations from that of the analyst who uses words to describe the psyche, although the two may at times overlap.

Scruton (2015) thinks that the aim of a poem is not to convince readers of the correspondence of words to reality. Rather, its purpose is to facilitate readers to imagine the world as the poet depicts.

Now, although poetic truth of this kind is not alien to psychoanalytic writings it does seem to differ from it in some important sense. Think about the authors of psychoanalytic texts. They all want first and foremost to understand an unknown psychic fact (empirical problem). The innovative analyst has just met a patient. This patient suddenly behaves in a strange way. The analyst is puzzled. She feels that she cannot rely on current theories. She is holding a curious not-knowing position until she discovers an interesting innovative explanation. She knows that her description must correspond to the psychic reality “out there” in the transference relations and that she cannot imagine it or make it up like a poet. The truth value of her innovative explanation depends not merely on how successfully it conveys experience (although this too is very important, see next section) but on how adequate her description is in convincing the community that it corresponds to psychic reality.

She is fully committed to something to which the poet is only loosely committed, namely to understand what it was that happened there – both in the past and in the present.

Despite their differences psychoanalytic writing and poetic writing have much in common. When a psychic fact is put into words the effect is sometimes emotionally powerful. And so psychoanalytic thinking, when it works well, can form a genre in its own right representing psychic facts in a singular way through the symbolic. Often, psychoanalytic innovations will describe universal themes – but always in singular terms. It is exactly this combination between the universal and the singular that is so powerful.

Here, to illustrate, is a passage from Parsons, on creativity:

If creativity is the discovery of what we had not thought of looking for, or the making of something which was, up till now, unimagined, it must call for a special sort of vulnerability. To open ourselves to the shock of creative discovery we must put ourselves at risk and be ready to give up, with no certainty about the future, ways of seeing which up till now have served us well (Parsons, 1990, p. 420).

Parsons does not only present an answer to a question (What are the conditions under which creativity flourishes? Answer: The ability to take risks, to forfeit certainty, and in other words: to be vulnerable); his writing also includes a poetic element (the surprising and metaphoric association between creativity, the hitherto unthought – and, on the other hand, vulnerability; as well as the musical-rhythmic effect of the juxtaposition of creativity-discovery-vulnerability-certainty).

The solution or answer and its psychoanalytic textual form, the scientific element and the poetic, cannot be seen in isolation. The emotional, musical qualities of the language add validity to the conceptualization.

Parsons could have put the same idea in many different forms, some of them a lot more scientific and purely informative. But it is not clear whether it would have passed had he not used this poetic mode of expression.

All psychoanalytic writing displays this tension: Scientific writing aims at solving empirical problems. It is disciplined, clear-cut, and tells us how to think. It is systematic and aims at solving profound mysteries and riddles. Poetic writing is undisciplined, unpredictable, and full of subjectivity; but it is passionate and spontaneous, seeking, rather, what Bollas calls “psychic intensities” (Bollas, 1995, p. 60). We need both, as they seem to empower each other, creating a certain energy that outweighs their separate meaning and impact.

### Fourth, Psychoanalytic Innovations Conceptualize What We Already Know

There is another sense in which innovation in science is unlike innovation in psychoanalysis. While new knowledge concerning a scientific problem reveals what we did not know earlier, psychoanalysis, like art, adds meaning to what we already knew. We can, in other words, regard psychoanalytic writing as a type of symbolic writing in so far as it takes an existing experience to which we had no verbal access and makes it accessible. Something we experienced without being aware acquires articulate meaning.

Both Meltzer and Bion emphasize that psychoanalysis has not discovered any new ideas about its subject, the human mind, and that it is unlikely to do so. But through the psychoanalytic method, old ideas can be rediscovered in a new context (Meltzer, 1983, p. 98).

And Freud writes:

“I find myself for a moment in the interesting position of not knowing whether what I have to say should be regarded as something long familiar and obvious or as something entirely new and puzzling” (Freud, 1940, p. 274).

The truth of Winnicott’s notion of transitional object as an object that is both real and also helps to maintain a connection to the absent mother is hence directly associated with the fact that a well-known and universal human experience here is formulated for the first time. When Klein spoke about manic defenses like contempt for the object and arrogance, she first put into words what we had known for a long time about our attitude to our closest objects. The psychic fact and the poetic are not two distinct modes of knowledge. The poetic supplies the esthetic experience to the real and rings with something of the mysterious truth we already knew.

While the clinical experience or phenomenon (envy, transitional object, slips of the tongue, castration experience) is what it is, in line with reality, its description can take many forms. This may be what characterizes psychoanalysis’ epistemology. Psychoanalytic innovation, from this perspective, occurs when new facts concerning a psychic phenomenon come to light, but our ability to perceive them as correct is based on the fact that we were already familiar with them. The poetic language in which a new description of a clinical phenomenon is couched functions like a muscle that supports its approach to the truth: it elicits a powerful emotional engagement with the newly discovered facts. We have experienced this knowledge, but it was unconceptualized so far. For the discovered facts to ring true, the poetic quality of the text is crucial. Should facts about the mind be communicated in terse scientific language, the reader would be unable to connect with the actually lived experience.

Loewald thought that language “ties together human beings and self and object world, and it binds abstract thought with the bodily concreteness and power of life” (Loewald, 1978, p. 204). This is because language, in the form of the sounds of mother’s speech, imbues the infant’s lived experience from the beginning of life. The sounds of mother’s speech are part of the infant’s experience of interacting with the mother, and over time, those sounds become differentiated from other sensations of the lived world as a special kind of sound; these special sounds grow into words. But the sounds also remain connected in memory to the rest of experience and, for that reason, a powerful way to recall one’s inner experience and communicate it to another. Indeed, the lived feeling that language can create is a reflection of its experiential nature. Although the semantic possibilities of words expand over development, they do not overtake the experiential possibilities. A word is always an experiential memory.

## Sensibilities: Second-Order Innovations

I first encountered the word “sensibility” in an article by Stolorow et al. (2001), authors who took a central role in developing the intersubjective approach. Inspired by postmodernism, this theory claimed that in the therapeutic encounter, an intersubjective domain arises in which two partners mutually constitute each other. This co-creation – rather than the isolated mind of the patient– is the heart of the therapy. They call this theory a “sensibility” rather than a clinical theory: their work, they claim, is informed by the clinician’s attitude and the ensuing process, rather than by any hard and fast procedure.

Psychoanalytic communities develop sensibilities when they turn outward: Methodological sensibilities absorb significant scientific changes (in brain science or in infant research); cultural-philosophical sensibilities incorporate changes in philosophy, culture, or society (e.g., constructivism, postmodernism, or feminism). Like a seismograph, they register these changes and bring them to bear in psychoanalysis. Introducing a foreign element to psychoanalytic discourse, texts of this kind are sometimes met with indifference or hostility. Studies based in brain or infant research seek to cast a different light on what exists, to change the approach to patients, rather than to change theoretical models. Both neuropsychoanalysis and infant research believe they are actually restoring something to psychoanalysis. Thus, infant researchers claim to only deepen analysts’ knowledge of the nature of early development which is, after all, so central to their theory. Similarly, neuropsychoanalysts think their findings expand the analyst’s therapeutic possibilities. They remind us that Freud himself was a neurologist and that he sought to integrate the study of the brain and psychoanalysis (Johnson and Flores Mosri, 2016).

## The Relational Sensibility – Truth, Knowledge, and Politics

The implications of the relational approach for the psychoanalytic encounter are far reaching. It assumes that the analytic relationship is systematically mutual and two-directional throughout. The notion of the therapist as object of the patient’s projected relations from the past is exchanged for one in which the therapist is a subject in the therapeutic relationship. Puget (2017) captures this difference in her use of “interference,” a mutual process, with which she replaces “transference,” which relies on notions of object-subject distinctness and wholeness.

The relational approach assumes that the therapist’s personal involvement in the therapy is inevitable. It is a matter of mobilizing this involvement for the patient’s benefit. Aron (1996) for instance argued that Freud’s elimination of the “subjective factor” in the therapeutic situation has been a damaging omission. Freud’s aim, in tune with the academic-scientific thinking of his times, was to achieve an objective science of the psyche. Postmodernism, as said, questioned this approach, by arguing that any theoretical model is historically and linguistically mediated. Thus, Hoffman (1998), a major proponent of the relational approach, argued that the patient’s experience in the therapy and the way it is subsequently made sense of, are understandings that emerge in the course of the encounter, based on both therapist’s and patient’s personal histories and their typical modes of organization. It would be inappropriate to judge these construals as simply wrong or right, rather than to think of them as more or less applicable.

A similar shift also occurred on the epistemological plane, where the relational approach no longer assumes one monolithic truth, embodied in a grand theory. Instead, theories are seen as possibilities, narratives that help in framing the therapeutic relationship and the patient’s psychic history and reality (Mills, 2005, 2017).

By redefining its boundaries of relevance, the relational approach has also come to include political issues that were outside the traditional scope of psychoanalysis. Clinical questions touching on gender and ethnicity are integral part of it, as well as for instance a critical attitude to the therapist’s unconscious racism. Altman writes:

“It should be taken for granted that none of us will be able to overcome our personal racist attitudes altogether. Thus, I am advocating that clinicians become familiar with their racism, not that they overcome their racist feelings and attitudes. The danger in implying that clinicians can and should overcome their racist feelings is that they will mistake their conscious goodwill and good intentions for a thoroughgoing nonracist attitude” (Altman, 2000, p. 601).

## A Sensibility Related to Infant Observations

Innovation also occurs when psychoanalysts are powerfully attracted to an extra-analytic domain of knowledge which while relating directly to psychoanalytic theory uses a different language. This extraneous knowledge, they believe, has the potential to influence the conceptualization and understanding of the therapeutic situation, however, in this particular case, rather than by reference to the adult therapeutic situation, through the observation of real interactions between infants and their caregivers. Infant researchers consider the method whereby infant development is derived from the psychoanalysis of adults or older children as naive.

Fonagy writes:

Melanie Klein’s baby and Winnicott’s baby were adultomorphic to a considerable degree. They seemed in some way put there to explain the conflicts and vicissitudes of the adult years rather than to genuinely map how the mind emerges from an early infant in many ways biologically and physically unprepared for the challenges of the external world. The infants of early psychoanalysis were retrofitted to the couch (Fonagy, 2014, p. xx)

The infant in these studies is extremely unlike the one featuring in Freud, Klein, Bion, Winnicott, and Kohut (Seligman, 2018). Traditional psychoanalytic theories present a baby with a primitive psychic organization which develops into an adaptive organization. Initially, this baby’s mental life is chaotic, unintegrated, and in conflict with the social world. Psychopathology is understood in terms of distortions in this process. The traces of psychosis or borderline personality can be discerned in early infancy.

Infant research has revealed a very different baby: This observed infant is not fundamentally disorganized, chaotic, or primitive, spending most of its time in a dream state or fearing persecution. Nor is it undifferentiated from its environment. From the very first moment, its life is organized around a relational matrix, which is continuously subject to reconstruction and change in line with experience (Stern, 1985). From day 1, the infant is already directed toward reality, influences and is influenced by its surroundings, is active and passive, dependent and independent, in possession of a variety of resources for organizing its behavior. One might have expected that anyone, once exposed to this research, would turn away from psychoanalysis – the chasm between these two conceptualizations is so enormous. But what actually happened was the opposite. The psychoanalytic infant research community, including researchers and practitioners who apply their insights in psychoanalytic psychotherapy, insists that its research is relevant to psychoanalysis and is bound to enrich it. They argue that their perspective is especially promising for work with adult patients who are more difficult to reach (Rustin, 2012).

In trying to understand the clinical distinction of this change in perspective, Stern observed that much of what occurs between people who are closely interacting involves an intersubjective consciousness. This implies that there is a realm of knowing that is implicit, outside awareness, and not requiring verbalization. The present moment, writes Stern (2004), rather than the past, becomes the focus. Stern (1985, 2004) extended this idea from everyday interaction to the patient/therapist encounter as crucial for an appreciation of how therapeutic change comes about. New ways of being and being with others are created through intersubjective processes of implicit relational knowing.

Rustin, who is identified with self-psychology, explains infant research’s complementary role vis a vis existing psychoanalytic theories very well in her book Infant Research and Neuroscience at Work in Psychotherapy (Rustin, 2012).

Because new facts about human behavior emerge daily psychoanalysts need to integrate these facts into their practice. She writes “These theories, and their accompanying principles and techniques, inform everything that I do. Through immersing myself in infant research, I found a way to expand and update self-psychology in my clinical practice” (p. 171). She argues that infant research’s focus on infant and caregiver relations underwrites Kohut’s notion of empathic engagement. But it also contributes to self-psychology by demonstrating that “an interaction is always a bidirectional, co-constructed process, thereby bolstering my commitment to intersubjectivity as a two-person model of clinical practice” (p. 172). Infant research, for Rustin, is not a substitute for any of the traditional theories or techniques, but it holds out opportunities for knowledge and intervention: “(…) additional sources of fluidity and elasticity to the therapeutic relationship and clinical process” (pp. 172–173).

Infant research posited a challenge to psychoanalytic methodology. Green (2000), who was fiercely critical of infant research, noted that infant research deviates from psychoanalysis’ investigation of the unconscious and the intrapsychic through the transference within the parameters set by the psychoanalytic setting. For analysts like him empirical researchers ignore the unconscious and change the psychoanalytic account into a theory of interpersonal relations.

Green even suggests that some researchers harbor sinister motivations, even those purporting to be acting in the interests of psychoanalysis: they want to get rid of psychoanalytic theories, in favor of a so-called scientific psychology, which is simpler, easier to teach, and more amenable to experimental studies. Green’s fears were exaggerated. Infat researchers aspired to make analysts sensitive to empirical findings, not to replace existing theories.

## A Sensibility Related to Neuropsychoanalysis

According to Johnson (2009), neuropsychoanalysis concerns the common ground between neuroscience and psychoanalysis. As said, neuropsychoanalysts claim that incorporating brain research into psychoanalysis is a continuation of Freud’s project. The neuropsychoanalytic community is very active and vibrant. It has founded an organization for its members, a journal, an annual conference, and research study groups all over the world.

Kaplan-Solms and Solms (2000) described contemporary use of neuroscience in psychoanalysis: “The aim of a depth neuropsychology is not to replace our psychic model of the mind with a physical one. Rather, our aim is to supplement the traditional viewpoints of metapsychology with a new, “physical point of view. The aim is to gain an additional perspective on something that can never be known directly” (p. 251).

It is plausible to think that this was appealing for the Neuropsychoanalysis community. Psychodynamic concepts such as cathexis, dreams, self, and ego may now appear in new light if we can trace their neurological underpinning.

For example, Northoff (2012) regarding the “self” suggest that instead of searching for the self-region or self-network in the brain, it will be more productive to search for essential executive and operational regularities. The spatiotemporal structure of the resting state may present the basis for such regularities. If it is linked with the self or the ego, one would assume the organization of the resting state’s spatiotemporal structure to somehow establish self-specific and thus to reveal the structure of the ego.

According to Yovell et al. (2015), for example, brain researchers discovered fear conditioning, a potent kind of implicit emotional learning. A person may have an extreme fear of a situation or another person without any explicit memory of why they have this fear. The corresponding implicit memory here is not repressed as classical psychoanalysis suggested. The authors argue that findings like this shed new light on a problem of traumatic memories that has haunted psychoanalysis from its first days. It explains why people who went through severe trauma were incapable to access direct memories of the traumatic events in spite of experiencing powerful, anxiety-inducing implicit memories.

Members of the neuropsychoanalysis community have published many other examples of how brain research can enrich and help analysts (Ruby, 2011; Giacolini and Sabatello, 2019; Iyengar et al., 2019). Some even have coined a new term “neuropsychoanalytic interpretation” (Johnson, 2009, p. 182).

Johnson, writes: “My definition of a’neuropsychoanalytic’ interpretation is a discussion of impingements on the patient’s thinking that clearly had to do with known biological factors. These included drug dreams, craving, justifications of using (clearly influenced by craving such as, “No one will know”), and telling her that craving would diminish with abstinence” (Johnson, 2009, p. 185).

In his case study he describes 28 “neuropsychoanalytic” interventions made in 60 h. These were the interpretations guided by his neurobiological knowledge regarding the patient’s addiction to cocaine such as identifying long-lasting transformations in brain structure and function that affected “the ventral tegmental-nucleus accumbens shell/dopamine-glutamate/hippocampal-amygdalar-cingulate-frontal functioning. The foremost manifestation of this change is the drug dream” (p. 186).

The “neuropsychoanalytic interpretation” also helped to ease shame. Johnson’s patient is enabled to understand that her behavior is not the outcome of some flaw in her personality.

He writes: “There is nothing remarkable or special about this treatment. It follows the ordinary psychoanalytic approach of having the patient free-associate and the analyst interpret. Comments about medication are required because they are part of the effort to facilitate her being sober” (p. 191).

According to Yovell et al., (2015), “psychoanalysis needs to be in a position to consider developments in science, even if it ends up dismissing some of them as irrelevant, due to the criteria and findings of its own epistemology” (p. 1528).

Like infant research and like the relational approach, neuropsychoanalysis challenges traditional psychoanalysis’ epistemology and methodology.

Johnson and Flores Mosri write:

“Neuropsychoanalysis is a twenty-first century development that has at its core the concept of dual aspect monism. Whether phenomena are evaluated empathically, or through measurements and statistics, it introduces an artifact of perception. Empirical data is filtered through the means by which it is made. Therefore, we are in the delightful position of being able to make observations by psychoanalytic clinical means that can also, perhaps with some technical difficulties, be made with genetic testing, animal observation of homologous behaviors, fMRI scanning, or some other nomothetic approach” (Johnson and Flores Mosri, 2016, pp. x).

## Can Sensibilities Address First-Order Questions?

I have portrayed the relational approach as a sensibility that poses second-order questions regarding existing theories, while the latter in turn discovers first-order psychoanalytic facts. Most relational analysts do of course have a keen interest in “facts” and “empirical problems” (see Govrin, 2006). Ghent (1990) explores what patients need and how this need unfolds in therapy; Davies and Frawley (1992) discovered new facts regarding patients who suffered from sexual abuse and Stern discovered a new component of the unconscious which he called “unformulated experiences” (Stern, 1983). There is no hard, binary division between these orders of questions: it is rather a matter of a tension between facts and sensibilities, and this exists not only between different psychoanalytic communities but also within each psychoanalytic orientation and within analysts themselves. This also is true for infant research and neuropsychoanalysis. They too are interested in how new facts can change the clinical practice.

And yet, there is a difference between sensibilities and first-order psychoanalytic facts. Postmodernists, especially the USA relational group, select what they consider valuable in existing theories, extricate it from its positivist moorings, and insert it into an intersubjective and constructivist model. Likewise, findings from neuropsychoanalysis and infant research offer a new and additional perspective, specifically on nonverbal and nonconscious processes of clinical interaction. The newer perspectives have opened creative pathways for empathic immersion, interaction, clinical understanding, and intervention. As Rustin (2012) writes: “What I do offer is a way to use these research findings as another lens through which to view or think about some nonverbal, nonconscious aspects of clinical data and to tailor interventions so that they more effectively lead to therapeutic action. I view these concepts as additions that increase the possibilities for spontaneous and imaginative ways of working with patients” (p. 10).

Psychoanalytic sensibilities are an attempt to integrate new disciplines and world views into mainstream clinical practice. Psychoanalytic schools, by contrast, have more ambitiously presented fully-fledged alternatives to already existing schools and offered new psychic facts concerning the link between infancy, psychopathology, and transference.

Rather than posing themselves as alternatives to psychoanalytic theories sensibilities try to engage in a dialogue. In neuropsychoanalysis, conscious id is another good case in point. Solms cites evidence that the upper brainstem (together with associated limbic structures) performs the functions that Freud attributed to the id, while the cortex (and associated forebrain structures) performs the functions he attributed to the ego. This is a radical new fact. It reveals a stark contradiction between the current concepts of affective neuroscience and those of Freud. The realization that Freud’s id is intrinsically conscious has implications for psychoanalysis, which Solms describes (for example, if the id is conscious what is unconscious is withdrawal of automatization processes).

Solms’s paper is a wonderful dialogue between brain research and Freudian theory. Solms goes back and forth from Freud’s writings to brain images and data to show where Freud was right and ahead of his time and where he erred. These illustrations show how neuropsychoanalysis and infant research mainly aim to shift the relations with the enduring metapsychology of psychoanalysis into something more workable.

Whereas psychoanalytic schools are all-encompassing and need no additions, the relational approach, infant research, and neuropsychoanalysis do not stand alone. They depend on a constant dialogue with psychoanalytic schools.

## Does Psychoanalysis Make Progress?

Now, I want to return to the questions I posed in the beginning of this paper: Does psychoanalysis make progress?

Let us start with “first-order questions “. If we accept Laudan’s (1977) philosophy of science, we appraise a theory by one sole criterion: whether it provides acceptable answers to significant questions. According to Laudan, problem-solving activity has no direct connections with truth, but this does not deprive the problem-solving model of its explanatory force. Rationality, argues Laudan, consists in doing or believing things because we have good reasons for doing so. What counts as a sound reason for accepting a new explanation in psychoanalysis? How can we know if a suggested description of, say, the dynamic of analyst-patient relations is a sound one?

It is important to note that any assessment of the rationality of accepting a particular theory is relative: it is relative to other earlier and competing theories, and to prevailing views on methodology and in the case of psychoanalysis, it is relative to the therapist’s own theoretical inclinations. So, a psychotherapist who prefers to base psychotherapeutic theory on evidence-based research will not find psychoanalytic solutions as effective. However, the solution can be satisfactory to a psychotherapist who perceives clinical observations and single case studies as valid sources of knowledge.

My own clinical experience demonstrates the power of psychoanalytic theory to solve puzzling empirical problems (see also Govrin, 2016). One of my patients, single and in his late thirties, presented significant progress. He had experienced years of harsh relations with people, periods of severe loneliness, and a lack of social relations. After several months in therapy, he seemed less guarded, his relations with his colleagues had improved, and for the first time, he had gained the approval of his directors. But the more he recovered, the more hostile he became to me and the more critical he grew of the therapy. He would joke at my lack of experience, mock my interventions, and complain about my inability to support him. When he was not in an aggressive mood, he would express his hopelessness feeling that I could not possibly help him. When he mentioned the improvements, he had made in his personal and professional life, he spoke with indifference, indicating that as far as he was concerned it had nothing to do with therapy. I was bewildered, ill at ease, and infuriated. It was odd: A person whose life had undoubtedly changed for the better because of therapy not only failed to acknowledge it as such but did everything he could to devaluate what had been attained. My patient’s attitude toward me was a clinical/empirical fact. It was not a matter of interpretation. Many psychotherapists, working in different orientations, have reported similar reactions.

My Kleinian supervisor helped me understand my patient’s inner dynamic and how it affected the transference relations. She explained how the Kleinian approach understands the phenomenon of the negative therapeutic reaction (NTR): Due to envy, which Klein maintained was the mental representation of the death instinct, the patient avoids any recognition of the goodness of the analyst so as to secure his omnipotent phantasy and deny his dependency. The libidinal force which directs him to love, show gratitude, and make amends—leading to a steady improvement of his condition—is overridden by envy, revenge, and contempt. My Kleinian supervisor added that the patient perceived “suffering” as the connection between us and could maintain contact with me only if he supposed we were both suffering.

I had no reason to doubt the accuracy of this “solution.” It seemed right and appropriate to this regressive phenomenon.

The literature on NTR is simply enormous. Analysts from different orientations have tried to explain it starting from Freud in his Wolf Man case study. Because numerous analysts have shared their thoughts and clinical experience the community obviously knows more about NTR than it did 50 years ago. Of course, the problem-solving effectiveness of the theories that explain NTR is not scientifically experimentally backed. But if a community of psychotherapists dealing with first-order questions use a strong set of psychological theories which they believe to be essential to the understanding of psychopathology, then it is perfectly rational to assess innovations, that is, “solutions,” in light of their capacity to be accommodated within that prior system of beliefs and assumptions. There is much more to say about the appraisal of effective “solutions” in psychoanalysis which is beyond the scope of this paper. This appraisal with its strange reliance on poetic style and the analysts’ subjectivity might seem odd for an outsider. Still, the psyche is “odd” so it is likely that the explanations will match its awkwardness. As Lear (1998) wrote:

“There is one way to refute psychoanalysis entirely: if from now on, every person would act rationally and clearly, it would be easy to dismiss psychoanalysis as idle chatter. However, since people often act in strange ways, causing pain to themselves and others, raising questions even among the players themselves, psychoanalysis will draw us to it” (p. 25).

Overall, I think that regarding first-order questions we are in a good position. NTR is just one example out of numerous “solutions” that psychoanalysts have found to puzzling psychic phenomena. Some of those problems (such as NTR) lack solutions from other nonpsychoanalytic theories (and other nonpsychoanalytic theories propose excellent solutions to other psychic first-order questions, though, I believe, in terms of scope, range and relevancy to human’s life psychoanalysis outnumber other theories).

Concerning second-order questions we must distinguish between the cultural – philosophical sensibility of the relational approach and the methodological sensibilities of neuropsychoanalysis and infant research. The relational approach, even if one resists its worldview, is no doubt the most important recent innovation in psychoanalysis. It revolutionized psychoanalysis by embracing new approaches to knowledge which led to a novel perception of therapeutic relations.

The relational approach first refers, rather to a whole new worldview according to which therapy is a genuine relationship between two persons and not merely some one-way internal relations that belong exclusively to the intrapsychic life of the patient’s mind (Davies, 1994, p. 168). The new worldview had important implications for analytic work (For example, how analysts work with enactments or the role of self-disclosure). As a result of this new worldview or, as I have been calling it, sensibility, analysts have changed how they think about their own subjectivity in the psychoanalytic encounter. This had put an end to therapists struggling to hide their personalities and blur their subjectivity in order to ensure patients’ emotional issues would stay untouched by the countertransference. Of course, this has taken its toll in the form of an indifference to theory (Govrin, 2006) or excessive use of analyst self-disclosure (Mills, 2017).

The epistemology of methodological sensibilities is very unlike that of the cultural-philosophical sensibilities of the relational approach. In fact, by relying on scientific research the epistemology of both neuropsychoanalysis and infant research is strikingly similar to that of the “silver era” of psychoanalysis where realism and correspondence theory of truth prevailed. So, contrary to the revolutionary meta-psychology of the relational approach, methodological sensibilities offer a new source of knowledge with a traditional epistemology. They challenge psychoanalytic sources of knowledge by offering to extend these sources by relying on other fields of knowledge besides the clinical encounter.

I believe that their greatest challenge is addressing an external conceptual problem (Laudan, 1977, p. 50). External conceptual problems are generated by a theory when it conflicts with another theory or doctrine which its supporters believe to be rationally well founded. There is a difference between mainstream psychoanalysis and between infant research and neuropsychoanalysis. For example, many mainstream analysts still use the term symbiotic phase despite findings from infant research showing that the infant from the very beginning is aware that she is physically separate, conscious of her caregivers and continuously relating to her surroundings. In science the answer to a “tension” between a methodology and a scientific theory is in many cases reached by changing the scientific theory as to adjust it to the methodological standards. In other instances, it is the methodology itself which is transformed. Neither of this had happened in psychoanalysis because of infant research and neuropsychoanalysis.

As a result, infant research and neuropsychoanalysis have not so far prompted a paradigm shift in psychoanalysis. Perhaps this is because neuropsychoanalysis has not yet reached a point of development that obliges mainstream analysts to consider it a serious contributor. It did not lead mainstream analysts to think that the inconsistency and correspondence between methodological sensibilities are convincing enough probably because they speak different languages and methodologies.

To the credit of researchers from both infant research and neuropsychaoanlysis, it must be said that they never thought that psychoanalysts should kneel before their own scientific findings because they are more grounded and evidence-based, nor have they expected analysts to abandon parts of mainstream psychoanalysis. I believe that psychoanalytic problem-solving effectiveness is improved by new insights from brain research and infant research through a process of inquiry, argument, and agreement within open-minded communities. And the reputation of psychoanalysis as a serious body of knowledge is enhanced if it can show that it is successful in incorporating current findings in science or at least conducts a healthy dialogue with these findings.

## Conclusion

I have demonstrated that any attempt to outline innovations in psychoanalysis will have to tread a narrow, dialectic line between two opposing directions. On the one hand, we can point out a dynamic of change in therapeutic approaches, the rise and fall of theoretical models, and the development of new therapeutic understandings of numerous psychic phenomena such as transference and psychopathology. On the other, there is the fact that there has not been all that much change in the influence of main psychoanalytic theory (Freud, Bion, Klein, Winnicott, Kohut, and others).

In science, we witness the same duality (Laudan, 1977). Some philosophers of science emphasize the radical shifts in scientific thought. Others stress the outstanding continuities that mark its evolution. I think we can learn from Laudan’s work on scientific progress and combine between the two perspectives within psychoanalysis when we think in terms of first and second order questions. Freud used a mechanistic-biological drive model to describe mental structures, object relations theories believe in self-object representations, whereas contemporary psychoanalysts perceive the analytic situation as shaped as a dynamic between two subjectivities. No doubt, this represents a movement of change in psychoanalysis. On the other hand, taking a “gradual” perspective, we may stress that psychoanalytic theory still champions Freud’s original profound link between psychopathology, past experience, and transference. We still listen to our patients’ unconscious, encourage them to free associate, interpret their dreams, consider unconscious transference dynamics as a decisive factor, and make the best interpretations that we think might help the patient to grow or improve his understanding of his inner motives.

The chief element of continuity in psychoanalysis (and in other sciences, see Laudan, 1977) is represented by the fundamental empirical problems or first-order questions. Ever since Freud every psychoanalytic school has addressed anxiety, psychosis, narcissism, perversions, regressions, dreams, and other psychic phenomena. Although the empirical problem domain varies (we see less hysterics and more personality disorders) as a result of cultural and social changes, psychic phenomena within the psychoanalytic encounter tend to endure.

Where radical shifts occur, it is not so much at the level of the formulation or identification of first-order problems as at the level of explanation or problem solution. There are, for example, radical differences between the way in which Kohut explains the parent-child relations and the way Freud did. But parent-child relations as such remain an essential problem for psychoanalysts. Of course, besides shared interest in the same psychic phenomena, there are often important common conceptualizations that persist through time (the central concept of transference, for example).

I assume this might seem strange to a contemporary relational analyst who perceives little if any contiguity between Freud’s drive theory, with its quasi-mechanistic and biological language, and the relational approach. A postmodernist would moreover add that psychoanalytic theories also and nevertheless leave their imprint on the first-order questions too. Hence, if the relational approach differs from Freud’s drive theory then all the terms within these theories must have different meanings.

However, even with using different epistemologies and methodologies, we still use different theories to explain the same problem (such as agoraphobia or psychosis) even when we describe the problem in different language.

In fact, I believe psychoanalysis’ merit is exactly in its ability to find effective explanations to significant psychic phenomena and clinical facts which are different from the psychoanalytic theories which attempt to solve them.

Psychic phenomena are therefore the “engine” behind psychoanalytic progress. In fact, this was the reason psychoanalytic schools have emerged from the first place. Each school defined different set of clinical phenomena that previous theories either overlooked or proposed an unsatisfactory explanation. Self-psychology covered empathy in development and clinical encounter; Klein covered paranoia and destructiveness, Kernberg covered borderline patients; Sullivan the interpersonal cultural dimension and so on.

This merit compensates for a methodology that relies on a clinical observation and on subjective theorizing, which thought by many as “crippling epistemological defect uncharacteristic of other science in that its theories are not subject to verification but must rely upon the point of view and basic assumptions of groups of analysts (Hanly, 1983, p. 402). One promising solution of how we can test our subjective theories, at least in the clinical encounter, was proposed by Hinshelwood (2013) which offered a “testing process” (p. 130) between different psychoanalytic theories based on occurrences that happen before and after interpretation.

Further inquiry needs to find how we distinguish between far-fetched and appropriate therapeutic solutions. We need to consider psychoanalytic schools in terms of their weakness and strength in finding effective “solutions” to psychic phenomena. Such inquiry can guide us to use different theories for understanding different facts.

## Author Contributions

The author confirms being the sole contributor of this work and has approved it for publication.

### Conflict of Interest Statement

The author declares that the research was conducted in the absence of any commercial or financial relationships that could be construed as a potential conflict of interest.

## Footnotes

^1^It is worth mentioning that no article by Freud appears in the top 3 most accessed or quoted articles. However, since 2009 Freud appeared in the title of 8,161 papers (compared to Klein 569, Winnicott 416, Bion 411 and Mitchell 132).
